# Recommendations for applying a multi-dimensional model of impulsive personality to diagnosis and treatment

**DOI:** 10.1186/s40479-018-0084-x

**Published:** 2018-04-02

**Authors:** Miji Um, Alexandra R. Hershberger, Zachary T. Whitt, Melissa A. Cyders

**Affiliations:** Department of Psychology, Indiana University – Purdue University Indianapolis, 402 N Blackford St, LD 124, Indianapolis, IN 46202 USA

**Keywords:** UPPS-P, Impulsive personality, Treatment, Diagnosis, DSM-5, Negative urgency, Lack of premeditation, Lack of perseverance, Sensation seeking, Positive urgency

## Abstract

The UPPS-P Model of Impulsive Personality, a prominent model of impulsive personality derived from the Five Factor Model of Personality, is a multi-dimensional model of impulsive personality that consists of negative urgency, lack of premeditation, lack of perseveration, sensation seeking, and positive urgency. The UPPS-P model has highlighted the importance of separating multidimensional traits due to the specificity of these traits corresponding to different risk behaviors. The goal of the current review paper is to make recommendations on how to apply the UPPS-P Model of Impulsive Personality, to diagnosis of and treatment for psychopathology. However, despite impulsivity being one of the most frequently used criteria for a number of clinical disorders, our review of the Diagnostic and Statistical Manual for Mental Disorders-5 found that the UPPS-P traits are not well represented in the diagnostic criteria, which we propose limits inferences about etiology and treatment targets. Additionally, research has largely focused on the importance of these traits for risk models; our review of the literature applying the UPPS-P traits to treatment processes and outcomes concluded that this area is not yet well studied. Here, we propose the specific application of the UPPS-P model to improve diagnosis and increase treatment effectiveness.

## Background 

Personality traits occur on a continuum ranging from normal to abnormal, and distinctions in personality patterns across the continuum correspond to therapeutically important differences in psychopathology. In the landmark study by Trull and Sher [1], patterns of personality traits by Five-Factor Model (FFM; i.e. Extraversion, Agreeableness, Conscientiousness, Neuroticism, Openness to Experience; [2]) aided in making important psychopathological distinctions; for example, discriminating between individuals with substance use disorders with and without depression. Thus, examining psychopathology through the lens of personality aids in differential diagnosis. Additionally, even for those diagnosed with the same disorder by the Diagnostic and Statistical Manual for Mental Disorders, 5th edition (DSM-5) [3], there can be significant variability in personality profiles between individuals. For example, Krueger and Eaton [4] highlighted multiple, distinct FFM personality profiles of individuals all diagnosed with Borderline Personality Disorder (BPD), with trait differences between individuals resulting in quite varied clinical presentations and conceptualizations. Thus, examining psychopathology through the lens of personality not only aids in distinguishing between disorders, but also provides individual level personality data within disorders, both of which are likely crucial in treatment planning, case conceptualization, and even rapport building.

The goal of the current review paper is to make recommendations on how to apply a prominent model of impulsive personality derived from the FFM, the UPPS-P Model of Impulsive Personality [5–7], to the diagnosis of and treatment for psychopathology. In this review, we first describe the history of the construct of impulsivity and how this led to the development of the UPPS-P multi-dimensional model of impulsive personality. We next provide a brief review how this model has been applied to risk assessment for psychopathology and associated maladaptive behaviors. We then report results of a qualitative review to summarize how impulsive personality is represented in DSM-5 diagnostic criteria. Our review found patterns of DSM-5 criteria that make it difficult to match criteria onto particular UPPS-P traits and we make suggestions to better bring the two into line. Finally, we report findings from both quantitative and qualitative reviews of the literature suggesting the importance of applying the UPPS-P traits to substance use treatment. In this section, we use substance use disorder as a prime example of how to apply the UPPS-P model to diagnosis and treatment, as the UPPS-P model has been most extensively researched in the substance use disorder literature. However, as Berg and colleagues [8] have shown, UPPS-P impulsive traits are implicated across a wide range of psychopathology, and we provide examples of clinical representation of impulsive personality in other DSM-5 disorders when appropriate.

## History of defining the construct of impulsivity

Impulsivity is comprised of two separate constructs: behavioral impulsivity and impulsive personality [9]. Behavioral impulsivity is measured using lab-task paradigms, such as the GoStop task and the Stop-Signal task [10], which are sensitive to rash action in the moment, while impulsive personality is most often assessed by self-report measures, which detect a tendency to behave or pattern of behaving impulsively. While both are associated with similar outcomes, such as substance use [11], meta-analytic findings have shown that the relationship between these constructs is weak (*r* = 0.10; [9]), most likely as a result of differences in measurement time course and methodology.

While conceptualizations of behavioral impulsivity and impulsive personality each have strengths and limitations, impulsive personality has advantages that make it an ideal construct from which to examine psychopathology. Impulsive personality has the advantage of content and ecological validity [12], reflecting cognitions, emotions, and behaviors individuals experience in everyday life. Some measures of behavioral impulsivity have been said to have content and ecological validity (e.g. two-choice impulsivity paradigm; [10]); however, the paradigms themselves do not easily generalize to real-world behaviors (e.g. Stop Signal Task; [10]) and only provide a snapshot of impulsive behavior at the time at which it is measured. Although impulsive personality measures are subject to self-report bias, interpreting them requires very little inference or assumption, particularly compared to behavioral measures, during which a number of complex neurobiological and psychological processes (e.g. eye-hand motor coordination, processing speed, familiarity with performing computerized tasks, and stereotype threat; see [13] for review) interact to produce the measurement outcome. As the goal of this review is to provide suggestions for improving diagnosis and treatment approaches, and as these are informed by general patterns of behavior more than specific instances of rash action, we choose to focus our discussion on impulsive personality. This decision is also supported by previous utility in applying FFM model of personality to psychopathology (e.g., [14]).

Modern discussions of impulsive personality arose out of early psychiatry and neuroscience. Perhaps the first well-known reference to impulsivity as a personality trait came from Esquirol, in his book *Treatise on Insanity* [15], in which he labeled a class of disorders “monomania,” a classification which closely resembled modern conceptualization of impulse control disorders. The neurologist and psychologist Ferrier [16] used the case of Phineas Gage to highlight distinct changes in impulsive personality following frontal lobe damage. Discussions of impulsivity as a personality construct continued to grow through the early twentieth century, with contributions made by Kraepelin [17], Freud & Brill [18], Bleuler [19], and Fenichel [20], all presenting impulsive personality as a maladaptive trait in the context of psychopathology (see [21] for a review).

Later, personality theorists began empirically deriving varying conceptualizations of what constitutes impulsive personality, as reviewed by Whiteside and Lynam [6]. For example, Eysenck and Eysenck [22] developed a model of personality that proposed impulsive personality is comprised of venturesomeness (related to extraversion) and impulsivity (related to psychoticism). Buss and Plomin [23] created a four-factor model of personality, proposing three facets of impulsive personality: the tendency to consider alternatives and consequences before making decisions, ability to remain with a task despite temptation, and the tendency to become bored and seek novel stimuli. Many other models of impulsive personality have been proposed, including Tellegen’s three-factor model [24, 25], Dickman’s two-factor model [26], Zuckerman’s model of sensation seeking [27], Cloninger’s model of novelty seeking [28, 29], Barratt’s three-factor model [30, 31], and three impulsive personality related scales of the NEO-PI-R [32]. Although there was some consensus across these measures (e.g., many included some aspect of sensation seeking or venturesomeness), there were more differences than commonalities. Given the transdiagnostic utility of the construct, and still no consensus on how it should be measured, researchers sought to construct a unified model of impulsive personality.

## Development of the UPPS-P model of impulsive personality

The original UPPS model was developed to provide consensus on which domains of impulsive personality were being assessed across measures. Whiteside and Lynam [6] used the FFM model of personality, which captures impulsive personality in four distinct facets (i.e., impulsiveness, self-discipline, excitement seeking, and deliberation; [2]) as a framework through which to construct a dimensional model of impulsive personality. To do this, Whiteside and Lynam [6] conducted a factor analysis of 10 existing impulsive personality measures in order to document underlying factors that would map onto the FFM model. Four facets of impulsive personality were extracted: *(negative) urgency*, or the tendency to act rashly while experiencing strong negative emotion, was associated with the impulsiveness facet of the FFM; *(lack of) premeditation*, or the tendency to act without thinking, was associated with the deliberation facet; *(lack of) perseverance*, or the tendency to become bored with and discontinue a task without finishing, was associated with the self-discipline facet; and *sensation seeking*, or the tendency to seek out novel exciting experiences, was associated with the excitement seeking facet. In 2007, Cyders and colleagues [7] developed the construct of *positive urgency*, or the tendency to act rashly while experiencing strong positive emotion, which was subsequently added to create the UPPS-P Impulsive Behavior Scale [5]. Although newer to the model, positive urgency was theorized to be unique due to anecdotal reports of positive emotion fueled celebratory events (e.g., vandalism in response to sports wins, flashing during Mardi Gras, drinking during celebrations) [33, 34].

Subsequent work has suggested the traits are best represented as a three-factor, hierarchical model, composed of 1) sensation seeking, 2) deficits of conscientiousness (with lack of planning and lack of perseverance as sub-factors), and 3) urgency (with negative and positive urgency as sub-factors) [35]. Interestingly, no model with an overall “impulsive personality” factor fit the data, suggesting that there is no single construct which we can point to as “impulsive personality,” so we should instead discuss these traits as separate, though related, factors [35]. Therefore, we refer to these traits as “UPPS-P traits” throughout the rest of this review.

The UPPS-P Impulsive Behavior Scale consists of 59 statements rated on a 1 to 4 Likert-type scale from “agree strongly” to “disagree strongly.” The UPPS-P questionnaire has been translated into more than 10 languages, including French [36], German [37], Italian [38], Spanish [39], Korean [40], Polish [41], Portuguese [42], and Dutch [43] and multiple short forms have been developed in Arabic [44], English [45], Farsi [46], French [47], German [48], Italian [49], and Spanish [50]. A child version also has been developed and validated [51]. To date, the original Whiteside and Lynam [6] article alone has been cited over 2500 times and the article describing subsequent development of positive urgency by Cyders and colleagues [7] over 600 times. Since its development, the UPPS-P Impulsive Behavior Scale has become a popular and useful tool for assessing multidimensional impulsive personality traits. These traits have been shown to relate to a wide range of psychopathology, including, but not limited to, substance use [52, 53], problem gambling [54, 55], risky sexual behaviors [56, 57], depression and anxiety [58], aggression [59, 60], borderline personality disorder [61], bipolar disorders [62, 63], suicidal and non-suicidal self-injurious behaviors [64, 65], and disordered eating [66, 67].

## Brief review of existing research with the UPPS-P model

Much of the work with the UPPS-P to date has focused on either how the UPPS-P traits are associated with existing psychopathology or else the extent to which they predict onset or increases in these symptoms over time. An important quantitative meta-analysis by Berg and colleagues [8] recently reviewed this vast literature and supported the specificity of the UPPS-P traits for psychopathology and maladaptive behaviors (i.e., alcohol and substance use, depression, suicidality and non-suicidal self-injurious behaviors, aggression, anxiety, BPD, and disordered eating). Interestingly, in this review, either positive or negative urgency showed the highest effects in each category of psychopathology reviewed, supporting the view that urgency in particular is a transdiagnostic endophenotype of risk [68], and consistent with other meta-analytic reviews [52, 69]. Negative urgency demonstrated the largest effect on every category of psychopathology reviewed (average effect size of *r* = .34 across disorders), except alcohol and substance use, where positive urgency demonstrated an effect of similar magnitude [8]. Lack of premeditation and lack of perseverance showed similar effect sizes across disorders, suggesting these traits could be equally important in diagnosis and treatment, for alcohol/substance use disorders, suicidality, and borderline personality disorder, although the authors discuss how these separate traits could be differentially contributing to aspects of disorders, such as Attention Deficit Hyperactivity Disorder [8]. Sensation seeking showed the most robust relationships with alcohol/substance use, suicidality, and aggression [8].

In addition, measurement of these five separate, though related, traits has allowed researchers to predict specific aspects of impulsive behavior within a particular disorder. One of the original and long-supported findings with the UPPS-P traits suggests the role of sensation seeking in the frequency of alcohol and drug use (including experimenting with new types of drugs), whereas negative urgency is related to problematic levels of use [52, 70]. Even though they are highly inter-correlated, differences in prediction between positive and negative urgency exist. For example, previous cross-sectional, longitudinal, and experimental emotion induction studies have demonstrated the distinctiveness of negative and positive urgency in risk-taking, such that negative urgency predicts negative emotion-based risk-taking while positive urgency predicts positive emotion-based risk-taking [7, 35, 54, 71, 72]. Additionally, only negative urgency is associated with binge eating [8, 69] and positive urgency is significantly greater among individuals with high risk for mania compared to those with low risk (negative urgency did not differ between groups) [63].

In sum, these data suggest that the UPPS-P traits demonstrate specificity in relation to psychopathology. The importance of this specificity should not be overlooked: in measuring a general disposition to impulsive personality, which lumps the various traits together, relationships with psychopathology may be masked or have their effect sizes diluted [73]. This idea is nicely demonstrated by Berg and colleagues [8]: For example, in the case of suicidality and non-suicidal self-injurious behavior, the averaged effect of UPPS-P traits is small and not significant (*r* = 0.18, *p* > .05), masking the larger effect of negative urgency on these behaviors (*r* = 0.25, *p* < .001). Similar masking effects are seen across the disorders reviewed by Berg and colleagues [8].

## Representation of UPPS-P traits in the DSM-5 diagnostic criteria

On the one hand, the association of UPPS-P traits with psychopathology should come as no surprise to those familiar with the DSM-5 [3], since impulsive behavior, defined broadly, is likely the most common diagnostic criterion apart from distress. However, identifying the specific traits underlying these criteria can prove difficult. In preparation for this manuscript, the authors reviewed all diagnostic criteria in the DSM-5 in order to identify how specific diagnostic criteria matched onto the UPPS-P traits. The first author (M.U.) evaluated each diagnostic criterion in the DSM-5, produced a “liberal” list of diagnostic criteria that seemed to represent UPPS-P model of impulsive personality traits, and designated initial coding. Then, the last three authors (A.R.H., Z.T.W., & M.A.C.) independently coded the list and provided the rationale. The codes were judged to be in agreement when at least three out of four authors agreed and in disagreement when the majority did not reach consensus.

In some cases, UPPS-P traits were clearly represented in DSM-5 diagnostic criteria (see Table 1). For example, negative urgency is represented in diagnostic criteria for posttraumatic stress disorder (e.g., *“Irritable behavior and angry outbursts (with little or no provocation) typically expressed as verbal or physical aggression toward people or objects.”*) and for gambling disorders (e.g., *“Often gambles when feeling distressed (e.g., helpless, guilty, anxious, depressed)”*). Positive urgency is represented in a diagnostic criterion for bipolar disorder (e.g., *“Excessive involvement in activities that have a high potential for painful consequences (*e.g.*, engaging in unrestrained buying sprees, sexual indiscretions, or foolish business investments) during the period of mood disturbance and increased energy or activity”*). Lack of perseverance is represented in diagnostic criteria for attention-deficit/hyperactivity disorder (e.g., *“Often has difficulty sustaining attention in tasks or play activities (e.g., has difficulty remaining focused during lectures, conversations, or lengthy reading)”*)*.* Lack of premeditation is represented in diagnostic criteria for substance use disorders (e.g., *“[substance] is often taken in larger amounts or over a longer period than was intended”*) and antisocial personality disorder (e.g., *“Impulsivity or failure to plan ahead”*). Sensation seeking is represented as a diagnostic criterion for borderline personality disorder (e.g., *“Impulsivity in at least two areas that are potentially self-damaging (*e.g.*, spending, sex, substance abuse, reckless driving, binge eating)”*).

**Table 1:** Representation of the UPPS-P model of impulsivity in DSM-5 disorder diagnostic criteria

Three-factor model	DSM-5 disorder	Diagnostic criteria [3] (Specific Traits when agreement was reached between raters)
Urgency	Posttraumatic Stress Disorder	E. Marked alterations in arousal and reactivity associated with the traumatic event(s), beginning or worsening after the traumatic event(s) occurred…: 1. Irritable behavior and angry outbursts (with little or no provocation) typically expressed as verbal or physical aggression toward people or objects. (Negative Urgency) 2. Reckless or self-destructive behavior (Negative Urgency)
	Intermittent Explosive Disorder	C. The recurrent aggressive outbursts are not premeditated (i.e., they are impulsive and/or anger-based) and are not committed to achieve some tangible objective (e.g., money, power, intimidation). (Negative Urgency)
	Bipolar I/II disorder	B. During the period of mood disturbance and increased energy or activity… are present to a significant degree and represent a noticeable change from usual behaviors: 7. Excessive involvement in activities that have a high potential for painful consequences (e.g., engaging in unrestrained buying sprees, sexual indiscretions, or foolish business investments). (Positive Urgency)
	Gambling disorder	A. Persistent and recurrent problematic gambling behavior leading to clinically significant impairment or distress…: 5. Often gambles when feeling distressed (e.g., helpless, guilty, anxious, depressed). (Negative Urgency)
	Borderline Personality Disorder	A. A pervasive pattern of instability of interpersonal relationships, self-image, and affects, and marked impulsivity…: 6. Affective instability due to a marked reactivity of mood (e.g., intense episodic dysphoria, irritability, or anxiety usually lasting a few hours and only rarely more than a few days). (Negative Urgency) 8. Inappropriate, intense anger or difficulty controlling anger (e.g., frequent displays of temper, constant anger, recurrent physical fights). (Negative Urgency)
Lack of conscientiousness	Attention-Deficit/Hyperactivity Disorder	A. A persistent pattern of inattention and/or hyperactivity-impulsivity that interferes with functioning or development…: 1. Inattention: …inconsistent with developmental level and that negatively impacts directly on social and academic/occupational activities: b. Often has difficulty sustaining attention in tasks or play activities (e.g., has difficulty remaining focused during lectures, conversations, or lengthy reading). (Lack of Perseverance) d. Often does not follow through on instructions and fails to finish schoolwork, chores, or duties in work place (e.g., starts tasks but quickly loses focus and is easily sidetracked). (Lack of Perseverance) f. Often avoids, dislikes, or is reluctant to engage in tasks that require sustained mental effort (e.g., schoolwork or homework; for older adolescents and adults, preparing reports, completing forms, reviewing length papers). (Lack of Perseverance)
	Intermittent Explosive Disorder	C. The recurrent aggressive outbursts are not premeditated (i.e., they are impulsive and/or anger-based) and are not committed to achieve some tangible objective (e.g., money, power, intimidation). (Lack of Premeditation)
	Kleptomania	A. Recurrent failure to resist impulses to steal objects that are not needed for personal use or for their monetary value (Lack of Perseverance)
	Substance use disorders	A. A problematic pattern of [substance] use leading to clinically significant impairment or distress…: 1. [Substance] is often taken in larger amounts or over a longer period than was intended. (Lack of Premeditation) 5. Recurrent [substance] use resulting in a failure to fulfill major role obligations at work, school, or home. 6. Continued [substance] use despite having persistent or recurrent social or interpersonal problems caused or exacerbated by the effects of alcohol. (Lack of Premeditation) 8. Recurrent [substance] use in situations in which it is physically hazardous. (Lack of Premeditation) 9. [Substance] use is continued despite knowledge of having a persistent or recurrent physical or psychological problem that is likely to have been caused or exacerbated by alcohol
	Gambling disorder	B. Persistent and recurrent problematic gambling behavior leading to clinically significant impairment or distress…: 6. After losing money gambling, often returns another day to get even (“chasing” one’s losses). (Lack of Premeditation) 8. Has jeopardized or lost a significant relationship, job, or educational or career opportunity because of gambling.
	Antisocial Personality Disorder	A. A pervasive pattern of disregard for and violation of the rights of others…: 3. Impulsivity or failure to plan ahead (Lack of Premeditation) 6. Consistent irresponsibility, as indicated by repeated failure to sustain consistent work behavior or honor financial obligations. (Lack of Premeditation)
Sensation seeking	Pyromania	C. Fascination with, interest in, curiosity about, or attraction to fire and its situational contexts (e.g., paraphernalia, uses, consequences). D. Pleasure, gratification, or relief when setting fires or when witnessing or participating in their aftermath.
	Gambling disorder	A. Persistent and recurrent problematic gambling behavior leading to clinically significant impairment or distress…: 1. Needs to gamble with increasing amounts of money in order to achieve the desired excitement
	Borderline Personality Disorder	B. A pervasive pattern of instability of interpersonal relationships, self-image, and affects, and marked impulsivity…: 4. Impulsivity in at least two areas that are potentially self-damaging (e.g., spending, sex, substance abuse, reckless driving, binge eating).
	Voyeuristic Disorder	A. …recurrent and intense sexual arousal from observing an unsuspecting person who is naked, in the process of disrobing, or engaging in sexual activity, as manifested by fantasies, urges, or behaviors.
	Exhibitionistic Disorder	A. …recurrent and intense sexual arousal from the exposure of one’s genitals to an unsuspecting person, as manifested by fantasies, urges, or behaviors
	Frotteuristic Disorder	A. …recurrent and intense sexual arousal from touching or rubbing against a nonconsenting person, as manifested by fantasies, urges, or behaviors.
	Sexual Sadism Disorder	A. …recurrent and intense sexual arousal from the physical or psychological suffering of another person, as manifested by fantasies, urges, or behaviors.
	Pedophilic Disorder	A. …recurrent, intense sexually arousing fantasies, sexual urges, or behaviors involving sexual activity with a prepubescent child or children (generally age 13 years or younger).
	Fetishistic Disorder	A. …recurrent and intense sexual arousal from either the use of nonliving objects or a highly specific focus on non-genital body part(s), as manifested by fantasies, urges, or behaviors
	Transvestic Disorder	A. …recurrent and intense sexual arousal from cross-dressing, as manifested by fantasies, urges, or behaviors

## Discordance between UPPS-P traits and DSM-5 diagnostic criteria

More often, however, we could not agree on the trait being referenced. There are some notable patterns in the points where we could not reach consensus. We believe these qualitative patterns can inform how the DSM-5 could better incorporate UPPS-P traits into disorder criteria.

First, although emotions are a central feature of many DSM-5 disorders, and negative and positive urgency have shown robust relationships to these disorders [8], the role of emotions and urgency are not clearly defined in the diagnostic criteria and little to no distinction is made between the roles of positive and negative emotions across disorders (see Table 2). Extensive research highlights the important role of emotions in DSM-5 disorders and maladaptive behaviors including, but not limited to, binge eating [74], substance use [75–77], depression [78–80], bipolar disorder [81, 82], obsessive compulsive disorder [83], BPD [84], schizophrenia [85–88], suicidal behavior [89], and aggression [59, 89]. However, current wording in many of the diagnostic criteria for these disorders does not directly address the role of emotions nor integrate the concepts of impulsive personality and emotions, thus precluding the research team from agreeing that the criterion represented the concept of urgency. Considering the fact that many well-known treatment approaches target emotion regulation to improve psychopathological symptoms (e.g., cognitive behavioral therapy targeting reduction in negative emotion through cognitive restructuring [90]; dialectical behavioral therapy enhancing emotion regulation and distress tolerance skills [91]; mindfulness therapy focusing on accepting but not reacting to feelings or thoughts [92]), it seems essential to incorporate the emotional aspects of clinical representation in the criteria for diagnosis.

**Table 2:** Disagreement in assigning an UPPS-P model of impulsivity trait in DSM-5 disorder diagnostic criteria

		Reason attributed to disagreement				
DSM-5 disorder	Diagnostic criteria [3]	Does not include emotion	Focus on wrong trait	Confounded by other constructs	Multiple traits in criteria	Compulsive or impulsive?
•Bipolar I/II Disorders •Major Depressive Disorder	A. …represent a change from previous functioning; at least one of the symptoms is either (1) depressed mood or (2) loss of interest or pleasure. 9. Recurrent thoughts of death (not just fear of dying), recurrent suicidal ideation without a specific plan, or a suicide attempt or a specific plan for committing suicide.					
				X		
Disruptive Mood Dysregulation Disorder	A. Severe recurrent temper outbursts manifested verbally (e.g., verbal rages) and/or behaviorally (e.g., physical aggression toward people or property) that are grossly out of proportion in intensity or duration to the situation or provocation.			X	X	
•Bulimia Nervosa •Binge Eating Disorder	A. Recurrent episodes of binge eating. An episode of binge eating is characterized by both of the following: 2. A sense of lack of control over eating during the episode (e.g., a feeling that one cannot stop eating or control what or how much one is eating).	X				
Oppositional Defiant Disorder	A. A pattern of angry/irritable mood, argumentative/defiant behavior, or vindictiveness…., and exhibited during interaction with at least one individual who is not a sibling.					
	1. Often loses temper.			X		
	4. Often actively defies or refuses to comply with requests from authority figures or with rules.			X		
Intermittent Explosive Disorder	B. Recurrent behavioral outbursts representing a failure to control aggressive impulses…					
	1. Verbal aggression (e.g., temper tantrums, tirades, verbal arguments or fights) or physical aggression toward property, animals, or other individuals…			X		
	2. The behavioral outbursts involving damage or destruction of property and/or physical assault involving physical injury against animals or other individuals…			X		
Conduct disorder	A. A repetitive and persistent pattern of behavior in which the basic rights of others or major age-appropriate societal norms or rules are violated…					
	2. Often initiates physical fights			X	X	
	3. Has used a weapon that can cause serious physical harm to others (e.g., a bat, brick, broken bottle, knife, gun).			X	X	
	6. Has stolen while confronting a victim (e.g., mugging, purse snatching, extortion, armed robbery)			X	X	
	7. Has forced someone into sexual activity			X	X	
	10. Has broken into someone else’s house, building, or car			X	X	
	12. Has stolen items of nontrivial value without confronting a victim (e.g., shoplifting, but without breaking and entering; forgery)			X	X	
	13. Often stays out at night despite parental prohibition, beginning before age 13 years.			X	X	
	14. Has run away from home overnight…while living in the parental or parental surrogate home…			X	X	
Pyromania	B. Tension or affective arousal before act			X		
Kleptomania	B. Increasing sense of tension immediately before committing the theft			X		
Substance Use Disorders	A. A problematic pattern of substance use leading to clinically significant impairment or distress…		X^a^			
	4. Craving, or a strong desire or urge to use [substance]	X				
	5. Recurrent [substance] use resulting in a failure to fulfill major role obligations at work, school, or home.					X
	6. Continued [substance] use despite having persistent or recurrent social or interpersonal problems caused or exacerbated by the effects of [substance].					X
	9. [substance] use is continued despite knowledge of having a persistent or recurrent physical or psychological problem that is likely to have been caused or exacerbated by [substance]					X
Gambling Disorders	A. Persistent and recurrent problematic gambling behavior leading to clinically significant impairment or distress… 5. Often gambles when feeling distressed (e.g., helpless, guilty, anxious, depressed).		X			
Antisocial Personality Disorder	A. A pervasive pattern of disregard for and violation of the rights of others… 1. Failure to conform to social norms with respect to lawful behaviors, as indicated by repeatedly performing acts that are grounds for arrest.			X	X	
	2. Irritability and aggressiveness, as indicated by repeated physical fights or assaults			X	X	
Borderline Personality Disorder	A. A pervasive pattern of instability of interpersonal relationships, self-image, and affects, and marked impulsivity…					
	5. Recurrent suicidal behavior, gestures, or threats, or self-mutilating behavior.			X		

*Notes*. ^a^ = negative and positive urgency is not represented in the diagnostic criteria for substance use disorder, and therefore, no specific diagnostic criteria are currently available to designate

For example, binge eating is a key characteristic of bulimia nervosa and binge eating disorder. Research has shown that negative emotions precede binge eating [74] and that negative urgency is an important risk factor [69]. A recent study showed that negative urgency is an important predictor of treatment outcome for binge eating disorder, such that greater negative urgency at baseline was related to a smaller reduction of binge eating frequency during and after treatment [93]. However, the emotional aspect of the binge eating is not recognized in DSM-5: “*A sense of lack of control over eating during the episode (e.g., a feeling that one cannot stop eating or control what or how much one is eating).*” Additionally, an important feature of substance use disorders is substance use associated craving (*“craving, or a strong desire or urge to use [the substance]”*) which is often induced by negative emotional states (and likely positive emotional states, although not well investigated) [94], and further, neurobiological evidence indicates negative urgency likely plays a causal role in substance craving [95, 96]. For clinicians, identifying underlying causes of craving, in this case emotional state, is imperative to developing a targeted approach to reducing craving; however, the DSM-5 does not specify the emotional basis of how or why cravings are triggered.

Second, in many cases the UPPS-P trait that is represented in diagnostic criteria does not match the trait that is best supported by research literature. This lead to difficulty of the authors to agree on which trait was or should be coded in the criteria. In substance use disorders, for example, lack of premeditation is well represented in the diagnostic criteria, (see Table 1) while negative and positive urgency is overlooked, despite their more robust relationships with more problematic and disordered substance use behaviors and consequences [8, 52, 97, 98]. This mismatch would lead to inadequate assessment, under-identification of individuals with urgency who are at higher risk, and mis-targeted treatment planning. Therefore, we suggest that negative and positive urgency would be important to include in the diagnostic criteria for substance use disorders, and that treatment should be customized based on whichever trait (whether it is negative and positive urgency or lack of premeditation) is driving the initiation, development or maintenance of disorder. Additionally, negative urgency is represented in the diagnostic criteria for gambling disorder. However, research indicates that there is a strong positive relationship between gambling behavior and positive urgency [7], and further, positive urgency uniquely predicts problematic gambling behavior, such as gambling with money that one cannot pay back [54]. Similar to substance use disorders, including both positive and negative urgency in the diagnostic criteria for gambling disorders is essential for interventions tailored to the specific urgency trait.

Third, representation of UPPS-P traits in some diagnostic criteria is often unclear or confounded by other constructs separate from, but related to, impulsive personality. This confounding of symptoms made it difficult for the authors to agree on the specific trait being represented, since the criteria include behaviors that may be driven by impulsive personality but could also be driven by other factors (see Table 2). For example, many of the defiant behaviors listed as diagnostic criteria for oppositional defiant disorder (e.g., *“Often actively defies or refuses to comply with requests from authority figures or with rules”*), conduct disorder (e.g., “*Has broken into someone else’s house, building, or car*”), and antisocial personality disorder (e.g., “*Failure to conform to social norms with respect to lawful behaviors, as indicated by repeatedly performing acts that are grounds for arrest*”) describe behaviors related to impulsive personality [61, 99], but these behaviors can also be purposeful and deliberate. Further, suicidal and non-suicidal self-injurious behaviors (e.g., “*Recurrent thoughts of death (not just fear of dying), recurrent suicidal ideation without a specific plan, or a suicide attempt or a specific plan for committing suicide*”) in bipolar disorder, BPD, or major depressive disorder are also often driven by impulsive personality [100], although some findings suggest “general impulsivity” to specifically predict suicide planning in adolescents with high risk for suicide [101, 102]. As written, the criteria do not specify if defiant or suicidal acts are committed willfully, in spite of expected outcomes, or whether they are done without considering the outcomes at all. Further, many of the diagnostic criteria used to describe aggressive or hostile behaviors (e.g., “*Often loses temper*” in oppositional defiant disorder; “*Irritability and aggressiveness, as indicated by repeated physical fights or assaults,*” in antisocial personality disorder) suggest the kind of emotional arousal associated with negative urgency, but could also be explained by cognitive factors such as hostile attribution bias; that is, a tendency to interpret ambiguous or neutral situations as threatening [103]. Two individuals then, with fundamentally different problems—cognitive error versus impulsive personality—will nevertheless qualify for the same diagnosis and likely be recommended the same treatment; however, less ambiguous criteria that distinguish impulsive personality traits from willful acts and cognitive errors might help steer an intervention beyond the presenting problem. Criteria that incorporate the intent and varying modes of decision making that give rise to problem behavior might suggest different etiological underpinnings and would dramatically impact the focus of intervention.

Fourth, many of the diagnostic criteria describe behaviors that could be loaded onto several UPPS-P traits, such that the criterion overall was not specific to a singular trait, making agreeing on the particular trait represented difficult (see Table 2). For example, in substance use disorders, the Criterion 4 “*Craving, or a strong desire or urge to use [substance]*” might indicate negative or positive urgency, or it could be sensation seeking driven. The Criterion 9 for substance use disorders specifies that *“[substance] use is continued despite knowledge of having a persistent or recurrent physical or psychological problem that is likely to have been caused or exacerbated by [substance],*” which could be attributed to both lack of premeditation and lack of perseverance. In disruptive mood dysregulation disorder, “*Severe recurrent temper outbursts manifested verbally (e.g., verbal rages) and/or behaviorally (e.g., physical aggression toward people or property) that are grossly out of proportion in intensity or duration to the situation or provocation*” also shows multiple impulsive personality traits including negative urgency and lack of premeditation. Further, many diagnostic criteria in conduct disorder present both sensation seeking and lack of premeditation (e.g., *“Often initiates fights,” “Has forced someone into sexual activity,”* and *“Has stolen while confronting a victim (e.g., mugging, purse snatching, extortion, armed robbery)”*). A single impulsive personality may be impacting problem behaviors; however, it could also be possible that multiple impulsive personalities are concurrently driving these behaviors. Thus, criteria distinguishing the impact of single impulsive personality or understanding the interactive effect of multiple impulsive personalities that give rise to these problem behaviors could suggest the focus and direction of treatment.

This lack of specificity is concerning for a number of reasons: To begin with, as noted above, each of the different UPPS-P traits has its own pattern of prediction associated with specific outcome risks (e.g., sensation seeking and binge drinking, or negative and positive urgency and drinking problems; first reported by [104] and further supported by a review by [52]). This specificity, however, is not reflected in DSM-5 criteria. For example, while lack of premeditation is well represented in the diagnostic criteria for substance use disorders, sensation seeking, negative urgency, and positive urgency are not. Furthermore, collapsing across the traits tends to mask and dilute the strength of effects, so using criteria that combine aspects, each with different levels of risk, impairs our ability to make predictions that influence how best to structure treatment [73]. In fact, this lack of specificity in criteria goes directly against the purported goal of the DSM-5 to promote further scientific knowledge concerning etiology and treatment of disorders, giving credence to the oft-cited criticisms of too much heterogeneity within disorders and too much overlap between disorders [73].

Fifth, a final point of confusion that emerged was the failure to distinguish impulsivity from compulsivity (see Table 2). The DSM-5 seems rather to integrate the two. For example, substance use disorders are characterized both by impulsivity in early stages of substance use and compulsivity later on [105]. In general, this diagnosis is seldom sought or given before the disorder has progressed to these later stages, when substance use is both on impulse and by compulsion. DSM-5 criteria for diagnosis, however, do not clearly tease the two apart. For example, “*Continued substance use despite having persistent or recurrent social or interpersonal problems caused or exacerbated by the effects of [substance],*” “*Recurrent [substance] use in situations in which it is physically hazardous,*” and “*[Substance] use is continued despite knowledge of having a persistent or recurrent physical or psychological problems that is likely to have been caused or exacerbated by [substance]*” represent both impulsivity (i.e., lack of premeditation) and compulsivity (i.e., persistent and persevering substance use in the face of adverse outcomes [106]). Although some features of the two are undoubtedly interconnected, it may serve clinicians better to have these constructs distinguished clearly, at least so that treatment design for substance use disorders will correspond with their developmental course.

We believe these patterns can inform how the DSM-5 could better incorporate UPPS-P traits into disorder criteria and we make a few potential recommendations here; however, we by no means see these recommendations as the only options for improvement. For instance, the Hierarchical Taxonomy of Psychopathology (HiTOP) Model has been recently proposed as a way to improve the reliability and validity of diagnosis and to reduce the heterogeneity within disorders, overlap between disorders, and diagnostic instability [107]. This movement proposes the use of a dimensional model of psychopathology through the lens of personality would alleviate many of the difficulties we report above, but also requires a large paradigm shift in diagnosis. This shift would greatly improve our ability to diagnose and treat psychopathology.

However, assuming that such a shift to the HiTOP Model might be slow to implement, we also make some general recommendations concerning improvements to the current DSM-5 diagnostic system that could relieve some of the problems we coded in our review. First, we suggest that DSM-5 criteria could better incorporate how emotions influence behavior, especially in cases where data are clear that impulsivity contributes to the disorder. Pertaining to our example above, a potential criterion for binge eating disorder could be rewritten to state “*Engages in uncontrolled eating in response to or during a negative affective state*.” Such a criterion would better capture the mood and impulsive components known to contribute to binge eating behaviors.

Second, we suggest that when writing diagnostic criteria, language should be avoided that confounds a particular impulsive personality trait with either other impulsive personality traits or other related constructs. As our example of substance use disorders notes, the criterion “*Craving, or a strong desire or urge to use [substance]*” could be attributed to positive or negative urgency, or even sensation seeking. This could be remedied by including wording to distinguish the idea of trouble resisting urgency (which would reflect urgency, e.g., “*Craving or a strong desire or urge to use [substance] that is difficult to resist*”) from the idea of seeking out new and exciting sensations (which would reflect sensation seeking, e.g., “*Craving or strong desire to use substances due to exciting properties of the drug experience*”).

Third, we suggest that the DSM-5 better match empirical data showing which UPPS-P traits are most highly implicated in the disorder in question. Pertaining to our example above, we suggest that substance use disorder criteria should reflect negative and positive urgency (e.g., “*Engages in drug use in response to negative or positive affect.*”) rather than lack of premeditation. We see the Berg and colleagues review [8] would provide a strong start to such an approach in determining which traits are important for disorders across the DSM-5. Finally, although mostly specific to substance use disorders, distinguishing between impulsive and compulsive behaviors would help inform the stage or severity of the disorder experienced by the individual.

Although the focus of the present paper is on the DSM-5, the issues we outline in Table 2 are likely echoed in the International Classification of Disease (ICD) System [108]. For example, ICD-10 diagnostic criteria for a depressive episode, similar to the DSM-5, includes suicide attempts (“*ideas or acts of self-harm or suicide”*) without specifying if the behavior is impulsive or planned. Additionally, similar to the DSM-5, diagnostic criteria for bulimia nervosa in the ICD-10 do not address the potential affective component of binge eating (*“the patient succumbs to episodes of overeating in which large amounts of food are consumed in short periods of time”*), in line with negative urgency, implicated in the etiology of the disorder. Thus, the recommendations we describe above in improving DSM-5 diagnostic criteria should also extend to the ICD-11, which is currently in preparation.

## Applying the UPPS-P traits to psychological treatment: a substance use treatment example

In order to review the extent to which UPPS-P traits are applied to psychological treatment, we conducted two complementary review processes. We focused these reviews on the application of the UPPS-P model in the treatment of substance use, as research in this area has been forefront in integrating the UPPS-P model to improve psychopathological symptoms. We use substance use as a prime example here, but we propose that a similar model could be applied to other disorders as well.

First, in order to examine the application of the UPPS-P traits to psychological treatment, we conducted a systematic quantitative review of the literature (as reported in [109]). The purpose of this meta-analysis was to quantitatively review existing work to examine 1) how impulsive personality affects substance use treatment outcomes and 2) how impulsive personality might change during substance use treatment. Articles were identified through: Key word searches in *Medline, PsychInfo, EMBase* and *PsychArticles,* based on an exhaustive combination of the following keyword groups: a) impuls*, sensation seeking, urgen*, persever* **or** premeditat*, b) substance, alcohol, drinking, heroin, opi*, *amphetamine, cocaine, stimulant, cannabis, **or** *marijuana,* and c) *treatment.* We also identified articles through e-mail alerts, reference sections of identified articles, forward searches of identified articles, and poster abstracts from the 2016 Research Society on Alcoholism Annual Meeting and Conference. Study authors were contacted in cases of missing information. Inclusion criteria for both study questions were: 1) report findings that contains some or all psychotherapy components and 2) report pre-treatment self-report measures of impulsive personality that map onto the UPPS-P framework [5] and are at least two items long.

For aim 1 (*k* = 12), significant effects were found for lack of premeditation (*g* = 0.60, *SE* = 0.30, *95% CI* 0.01 to 1.20; *z* = 1.99, *p* = .05) and negative urgency (*g* = 0.55, *SE* = 0.17; *95% CI* 0.22 to 0.88, *z* = 3.30, *p* = .001), with higher impulsive personality trait scores related to poorer substance use treatment outcomes. For aim 2 (*k* = 14), changes in sensation seeking (*g* = − 0.10, *SE* = 0.05, *95% CI* -0.20 to 0.004; *z* = − 1.88, *p* = .06) and negative urgency (*g* = − 0.25 *SE* = 0.14, *95% CI* -0.53 to 0.03; *z* = − 1.75, *p* = .08) approached significance. Overall, this meta-analytic review found that lack of premeditation and negative urgency are related to poorer substance use treatment outcome. Although negative urgency and sensation seeking are changing during treatment, the magnitude of the change is quite small, likely contributing to poor treatment outcomes and relapse.

Second, we qualitatively reviewed the literature to determine patterns in how the UPPS-P traits are currently represented in substance use treatment (Table 3). We conducted a systematic review of the literature to identify studies that specifically assessed changes in impulsive personality traits pre to post treatment using interventions outlined by Zapolski and colleagues [110]. Studies were identified using *Medline, PsychInfo, EMBase, PsychArticles* and *GoogleScholar.* Articles published through August 2017 were chosen based on an exhaustive combination of the following keyword groups: 1a) impuls*, sensation seeking, urgen*, persever* or premeditat* or 1b) *UPPS-P,* and 2) *treatment* or *intervention.* Studies were included in Table 3 if they 1) assessed changes in impulsive personality traits using the UPPS-P model or trait(s) that map onto the UPPS-P framework (see [9] for a review), and 2) reported administering an intervention in line with recommendations by Zapolski and colleagues’ [110] (see Table 3). Our search yielded *N* = 17 studies that met inclusion criteria. Identified studies were then coded for sample type, intervention used, and changes in impulsive personality traits pre to post treatment (statistically significant increase, decrease, or no change). The first author (M.U.) initially coded the identified studies, and the second author (A.R.H.) confirmed the coding determined by the first author.

**Table 3:** The UPPS-P model specific interventions and current empirical supports for treatment-related changes in impulsivity

Trait	Proposed intervention in Zapolski et al. [110]	Empirical findings	Sample	Interventions used	Change in impulsivity pre to post treatment
Negative urgency	Emotion regulation, distress tolerance, interpersonal effectiveness	Axelrod, Perepletchikova, Holtzman, & Sinha [84]^a^	Female outpatients with BPD & substance dependence	DBT	↓
		Weiss et al. [124]	Female college students	Emotion modulation	↓
		Zapolski & Smith [125]	Middle school youth experiencing behavioral or academic problems	DBT skills group	↓
	Adjust emotional reactions by considering the context, experience the emotion without acting, adjust reactions through relaxation, prayer, and other soothing activities, learn to effectively communicate feelings to others	Amaro et al. [126]^a^	Female Hispanic inpatients with drug addiction and co-occurring mental health disorders	Spiritual Self-Schema (Mindfulness & harm reduction)	–
		Margolin et al. [127]^a^	Drug users with HIV enrolled in a methadone maintenance program	Spiritual Self-Schema (Mindfulness & harm reduction)	↓
		Littlefield et al. [128]	Inpatients from a residential substance use disorder treatment facility	12-step group, CBT, DBT, MI	↓
		Reis, Castro, Faria, & Laranjeira [129]^a^	Male outpatients with cocaine dependence	Assertive strategic counseling & topiramate	–
		Blonigen, Timko, Moos, & Moos [130]^a^	Treatment-naïve individuals with alcohol use disorders	AA	↓
	SSRIs	Rinne, van den Brink, Wouters, & van Dyck [131]^a^	Females with BPD	Fluvoxamine	–
	Identify precipitating events or triggers to emotional reactivity and learn adaptive alternatives similar to those provided in distress tolerance modules	Santos-Ruiz, Robles-Ortega, Pérez-García, & Peralta-Ramírez [139]	Individuals with perceived high stress levels	CBT for stress management	↓
	Learn to evaluate behavioral choices in terms of one’s long-term goals	X			
Positive urgency	Teach adaptive techniques for savoring success and positive mood	X			
	Identify alternative, safer means of celebrating	X			
	Learn to use cues indicating risk for maladaptive behavior	X			
	Provide client with reminders or cues of the alternative behaviors identified	X			
Sensation seeking	Highly stimulating media messages suggesting alternative, safe ways to pursue stimulation	X			
	Development of a bank of safe, stimulating activities as behavioral options	X			
Lack of Premeditation	Cognitive mediation training (anticipating both positive and negative consequences of possible actions)	Weiss et al. [124]	Female college students	Impulsivity reduction	↓
	Specifying all steps necessary to complete a task and the time necessary for each step	Kendall & Finch [132]^a^	Children identified as impulsive	Verbal self-instructions & response-cost contingency	↓
		Kendall & Wilcox [133]^a^	Children with classroom interference behaviors	Verbal self-instructions, response-cost contingency & psychoeducation	–
	Learn to anticipate the consequences of one’s presence in situations and settings	Amaro et al. [126]^a^	Female Hispanic inpatients with drug addiction and co-occurring mental health disorders	Spiritual Self-Schema (Mindfulness & harm reduction)	–
		Margolin et al. [127]^a^	Drug users with HIV enrolled in a methadone maintenance program	Spiritual Self-Schema (Mindfulness & harm reduction)	↓
		Aklin, Tull, Kahler, & Lejuez [134]^a^	Inpatients admitted to residential substance use treatment facility	AA/NA, relapse prevention & functional analysis	–
		Gonçalves et al. [135]^a^	Inpatients enrolled in cocaine dependence treatment	Motivational Chess	–
Lack of perseverance	Stimulant medications plus cognitive-behavioral therapy	X			
	Behavioral paradigms to reinforce task completion	X			
	Learn to gauge attention span and distractibility delay, modify environment, learn techniques to reduce procrastination and increase follow-through	X			

*Notes*. *BPD* Borderline personality disorder, *DBT* Dialectical behavior therapy, *AA/NA* Alcoholics Anonymous/Narcotics Anonymous

↓ Statistically significant decreases in impulsivity pre to post treatment

– Non-significant change in impulsivity pre to post treatment

X = No empirical support available to the authors’ knowledge

^a^ indicates studies did not use UPPS-P Impulsive Behavior Scale

Given the body of literature implicating UPPS-P traits with a range of clinical disorders and problems [8, 52, 97, 111–113], it is somewhat surprising that comparably little has been done in applying the UPPS-P model to clinical practice in a systematic way. Our review of this research led us to conclude that data examining how UPPS-P traits interact with treatment processes and outcomes are limited to date; therefore, it may have seemed premature to begin applying the UPPS-P model to clinical practice. At the same time, extensive theory and empirical data support the transdiagnostic risks associated with UPPS-P traits. We therefore believe that measuring UPPS-P traits before and over the course of treatment can significantly aid clinicians in identifying specific targets for intervention; and further, lead to the development of novel treatment approaches which target the UPPS-P traits in particular.

Many existing treatments target proximal factors of clinical problems, rather than the UPPS-P traits that underlie them. Often, these proximal factors mediate the relationship between UPPS-P traits and the clinical disorder. For example, one way in which impulsive personality influences substance use is by affecting how a person learns about the behavior. Such traits make it more likely for an individual to form more positive beliefs or expectancies related to substance use, which in turn fuel further use [114]. Many psychotherapies for substance use disorders target these more proximal substance use motives, beliefs or expectancies, and self-efficacy, and research has established that UPPS-P traits are related to substance use through these factors [115–117]. It is thus possible that more distal factors, particularly UPPS-P traits, are left unchanged and potentially leave individuals at risk for symptom relapse or treatment non-response. Taken together, this literature led us to believe that more attention should be paid to UPPS-P traits in treatment assessment and development, as this could likely improve treatment outcomes.

Despite the wealth of literature implicating the UPPS-P model of impulsive personality in the development and maintenance of substance use disorders [118–120], minimal work has examined if decreases in UPPS-P traits are related to better substance use treatment outcomes (e.g. less frequent use, increased global functioning). Some treatments have aimed to directly target sensation seeking and “general impulsivity” in youth [121–123]. This is a promising step; however, findings provide little to no data to determine if changes in sensation seeking or general impulsive traits served as a mechanism for substance use change. A recent meta-analysis by Hershberger and colleagues [109] found that 1) lack of premeditation and negative urgency at intake are significantly related to poorer substance use treatment outcomes (*g’s* 0.60 and 0.55, respectively), and 2) although sensation seeking and negative urgency show significant decreases pre to post substance use treatment, these decreases are small (*g*’s 0.10 and 0.25, respectively). Taken together, UPPS-P traits appear to impart risk for poor treatment outcomes, and further, are not changing greatly through the course of substance use treatment. It thus behooves researchers and clinicians to follow-up on and clarify these findings by determining if changes in UPPS-P traits are potential mechanisms of substance use change across treatment.

Some work has aimed to specifically target UPPS-P traits with treatment design. Zapolski, Settles, Cyders, & Smith [110] proposed treatment strategies that target specific impulsive personality traits according to the UPPS-P model, and many of these strategies have been tested and supported (see Table 3). In Table 3, we present each UPPS-P trait, specific interventions suggested by [110], and empirical data (or lack thereof) showing whether they resulted in reductions of the trait. The majority of studies have focused on negative urgency, examining changes in the trait pre to post intervention. Across reviewed studies (Table 3) [84, 124–131], the majority of interventions showed reductions in negative urgency, although three studies failed to find significant changes [126, 129, 131]. For lack of premeditation, three studies demonstrated reductions pre to post treatment [124, 127, 132], while four studies failed to find significant change [126, 133–135].

Our review of the literature did not produce studies that examined changes in sensation seeking, positive urgency, or lack of perseverance through treatment recommendations proposed by [110]. For sensation seeking, interventions that directly target this trait do exist, but these studies failed to examine changes in sensation seeking itself [121, 122, 136]. While the theoretical orientation of the intervention is different, behavioral activation interventions could be considered as addressing the proposal made by [110] in regards to positive urgency (e.g., identifying alternative, safer means of celebrating) or sensation seeking (e.g., development of a bank of safe, stimulating activities as behavioral options). Treatments targeting persistence toward goals [137] could address lack of perseverance.

Together, information on the effectiveness of interventions reducing UPPS-P traits is sparse, and findings somewhat mixed. This is problematic, given the clear role these traits play in the development, maintenance, and persistence of a multitude of clinical disorders and problems (e.g., [8, 52, 97, 98, 109]). Further, even across studies that showed reductions in, for example, negative urgency, a wide array of treatment modalities was used, including Dialectical Behavioral Therapy, 12-step groups, and Cognitive Behavior Therapy. This makes it difficult to ascertain the mechanism of change; and further, makes it difficult for clinicians and researchers to replicate reductions in negative urgency. In some studies that we reviewed (see Table 3), specific traits were targeted, but impulsive personality was assessed using measures that do not assess the relevant traits; an oversight which doubtless introduced some imprecision in capturing treatment effects for a given impulsive trait. Future studies would benefit by assessing impulsive traits that the intervention designs are supposed to impact.

Although impulsive personality is a multi-faceted and multi-dimensional construct, current treatment options that target impulsive personality focus on only a few traits. As such, novel interventions are needed to target the less studied traits. For example, future studies can incorporate treatments suggested by [110] to target positive urgency, sensation seeking, or lack of perseveration (See [110] for details). Developing treatments targeting positive urgency seems especially important, especially given its robust effects on a wide range of psychopathology (similar in magnitude to negative urgency) [8].

## Conclusion

Since its inception, the UPPS-P model of impulsive personality has improved the prediction of psychopathology [8] and shown specificity of traits to corresponding risk behaviors [52, 97]. Although “impulsivity,” generally defined, is highly represented in clinical disorder criteria, our review of the DSM-5 diagnostic criteria concluded that most criteria are not written to map well onto specific UPPS-P traits. Our review also concluded that although the application of the UPPS-P traits to treatment is still in its infancy, recent work does suggest that specific UPPS-P traits negatively influence the effectiveness of substance use treatment response [109, 138].

In this review, we propose that the empirical data supporting the roles for discrete UPPS-P traits in a wide range of psychopathology and maladaptive behaviors have not yet been well integrated into DSM-5 disorder criteria, and that this limitation is an impediment to our understanding of etiology and treatment planning. We also propose that viewing psychopathology through the lens of the UPPS-P model will improve diagnosis and treatment. We review how specific UPPS-P traits may impede treatment effectiveness and may leave one at risk for relapse post treatment, using substance use as a prime example, although we propose that effects are likely more generally applicable to other forms of psychopathology. We make specific suggestions on how to target UPPS-P traits in treatment, which should be examined empirically. At the very least, we suggest that both researchers and clinicians should assess and track UPPS-P traits in treatment and clinical research. A better option is to also target specific traits during treatment, according to the empirical evidence supporting their role in that given clinical disorder or problem. Given the ease of access and implementation of the UPPS-P scale (including the availability of short forms and multiple translations, see www.impulsivity.org/measurement/UPPS_P), this is an accessible goal for researchers and clinicians alike. Many of the proposed treatments to target UPPS-P traits could also be easily implemented with or in addition to other empirically supported treatments, making this a low-cost, feasible, and potentially high impact strategy to improve clinical outcomes.

## Abbreviations

BPD: Borderline personality disorder
DSM-5: Diagnostic and statistical Manual for mental disorders, 5th edition
FFM: Five-factor model
HiTOP: Hierarchical taxonomy of psychopathology
ICD: International classification of disease

## References

[1] Trull TJ, Sher KJ (1994). Relationship between the five-factor model of personality and Axis I disorders in a nonclinical sample. J Abnorm Psychol.

[2] Costa PT, McCrae RR (1990). Personality disorders and the five-factor model of personality. J Personal Disord.

[3] APA (2013). Diagnostic and statistical manual of mental disorder.

[4] Krueger RF, Eaton NR (2010). Personality traits and the classification of mental disorders: toward a more complete integration in DSM-5 and an empirical model of psychopathology. Pers Disord Theory Res Treat.

[5] Lynam DR, Smith GT, Whiteside SP, Cyders MA (2006). The UPPS-P: Asessing five personality pathways to impulsive behavior.

[6] Whiteside SP, Lynam DR (2001). The five factor model and impulsivity: using a structural model of personality to understand impulsivity. Pers Individ Dif..

[7] Cyders MA, Smith GT, Spillane NS, Fischer S, Annus AM, Peterson C (2007). Integration of impulsivity and positive mood to predict risky behavior: development and validation of a measure of positive urgency. Psychol Assess.

[8] Berg JM, Latzman RD, Bliwise NG, Lilienfeld SO (2015). Parsing the heterogeneity of impulsivity: a Meta-analytic review of the behavioral implications of the UPPS for psychopathology. Psychol Assess.

[9] Cyders MA, Coskunpinar A (2011). Measurement of constructs using self-report and behavioral lab tasks: is there overlap in nomothetic span and construct representation for impulsivity?. Clin Psychol Rev.

[10] Dougherty DM, Mathias CW, Marsh DM, Jagar AA (2005). Laboratory behavioral measures of impulsivity. Behav Res Methods.

[11] Sharma L, Markon KE, Clark LA (2014). Toward a theory of distinct types of “impulsive” behaviors: a meta-analysis of self-report and behavioral measures. Psychol Bull.

[12] Sperry SH, Lynam DR, Walsh MA, Horton LE, Kwapil TR (2016). Examining the multidimensional structure of impulsivity in daily life. Pers Individ Dif..

[13] Argyriou E, Um M, Carron C, Cyders MA (2018). Age and impulsive behavior in drug addiction: a review of past research and future directions. Pharmacol Biochem Behav.

[14] Widiger TA (2011). Personality and psychopathology. World Psychiatry.

[15] Esquirol E. Mental maladies; a treatise on insanity: Lea and Blanchard; 1845.

[16] Ferrier D (1878). The Goulstonian lectures on the localisation of cerebral disease (concluded). Br Med J.

[17] Kraepelin E (1904). Lectures on clinical psychiatry.

[18] Freud S, Brill AA. Three contributions to the theory of sex (no. 7). New York: Nervous and mental disease publishing company; 1920.

[19] Bleuler E, Brill AA (1924). Textbook of psychiatry.

[20] Fenichel O (1945). Neurotic acting out. Psychoanal Rev.

[21] Moeller FG. In: Grant JE, Potenza MN, editors. Historical perspective on impulsivity and impulse control disorders: The Oxford handbook of impulse control disorders. Oxford University Press; 2011. p. 11–21.

[22] Eysenck SB, Eysenck HJ (1978). Impulsiveness and venturesomeness: their position in a dimensional system of personality description. Psychol Rep.

[23] Buss AH, Plomin R (1975). A temperament theory of personality development.

[24] Tellegen A. Brief manual for the multidimensional personality questionnaire. Unpublished manuscripts. Minneapolis: University of Minnesota; 1982.

[25] Tellegen A. In: Tuma AH, Maser JD, editors. Structures of mood and personality and their relevance to assessing anxiety, with an emphasis on self-report: Anxiety and the anxiety disorders. Erlbaum; 1985. p. 681–706.

[26] Dickman SJ (1990). Functional and dysfunctional impulsivity: personality and cognitive correlates. J Pers Soc Psychol.

[27] Zuckerman M (1991). Psychobiology of personality (Vol. 10).

[28] Cloninger CR, Przybeck TR, Svrakic DM (1991). The tridimensional personality questionnaire: US normative data. Psychol Rep.

[29] Cloninger CR, Svrakic DM, Przybeck TR (1993). A psychobiological model of temperament and character. Arch Gen Psychiatry.

[30] Barratt ES, Patton J, Stanford M. Barratt impulsive scale. Texas: Barratt-Psychiatry Medical Branch, University of Texas; 1975.

[31] Patton JH, Stanford MS, Barratt ES (1995). Factor structure of the Barratt impulsiveness scale. J Clin Psychol.

[32] Costa PT, McCrae RR. Revised NEO personality inventory manual. Odessa: Psychological Assessment Resources; 1992.

[33] White A, Hingson R (2013). The burden of alcohol use: excessive alcohol consumption and related consequences among college students. Alcohol Res.

[34] Del Boca FK, Darkes J, Greenbaum PE, Goldman MS (2004). Up close and personal: temporal variability in the drinking of individual college students during their first year. J Consult Clin Psychol.

[35] Cyders MA, Smith GT (2007). Mood-based rash action and its components: positive and negative urgency. Pers Individ Dif..

[36] Van der Linden M, D’Acremont M, Zermatten A, Jermann F, Laroi F, Willems S (2006). A French adaptation of the UPPS impulsive behavior scale: confirmatory factor analysis in a sample of undergraduate students. Eur J Psychol Assess.

[37] Kampfe N, Mitte K (2009). A German validation of the UPPS impulsive behavior scale: further evidence for a four-dimensional model of impulsivity. Eur J Psychol Assess.

[38] Fossati A, Somma A, Karyadi KA, Cyders MA, Bortolla R, Borroni S (2016). Reliability and validity of the Italian translation of the UPPS-P impulsive behavior scale in a sample of consecutively admitted psychotherapy patients. Pers Individ Dif.

[39] Verdejo-García A, Lozano Ó, Moya M, Alcázar MÁ, Pérez-García M (2010). Psychometric properties of a Spanish version of the UPPS-P impulsive behavior scale: reliability, validity and association with trait and cognitive impulsivity. J Pers Assess.

[40] Lim SY, Lee YH (2014). A Korean validation of the UPPS-P impulsive behavior scale in college students. Korean J Clin Psychol.

[41] Poprawa R (2016). Polish adaptation of the UPPS-P impulsive behavior scale and its significance in the prediction of selected externalized problems and disorders. Przegląd Psychol.

[42] Sediyama CYN, Moura R, Garcia MS, da Silva AG, Soraggi C, Neves FS (2017). Factor analysis of the Brazilian version of UPPS impulsive behavior scale. Front Psychol.

[43] Bousardt AMC, Noorthooorn EO, Hoogendoorn AW, Nijman HLI, Hummelen JW. On the link between emotionally driven impulsivity and aggression: evidence from a validation study on the Dutch UPPS-P. Int J Offender Ther Comp Criminol. 2017:1–16. http://journals.sagepub.com.proxy.ulib.uits.iu.edu/doi/pdf/10.1177/0306624X17711879. 10.1177/0306624X17711879 28569075

[44] Bteich G, Berbiche D, Khazaal Y (2017). Validation of the short Arabic UPPS-P impulsive behavior scale. BMC Psychiatry..

[45] Cyders MA, Littlefield AK, Coffey S, Karyadi KA (2014). Examination of a short English version of the UPPS-P impulsive behavior scale. Addict Behav.

[46] Shokri O, Sanaeeour MH (2016). Cross-cultural adaptation of a Farsi version of the impulsive behavior scale – short form in Iran. Int J Body, Mind Cult.

[47] Billieux J, Rochat L, Ceschi G, Carré A, Offerlin-Meyer I, Defeldre AC (2012). Validation of a short French version of the UPPS-P impulsive behavior scale. Compr Psychiatry.

[48] Keye D, Wilhelm O, Oberauer K (2009). Structure and correlates of the German version of the brief UPPS impulsive behavior scales. Eur J Psychol Assess.

[49] D’Orta I, Burnay J, Aiello D, Niolu C, Siracusano A, Timpanaro L (2016). Development and validation of a short Italian UPPS-P impulsive behavior scale. Addict Behav Rep.

[50] Candido A, Orduna E, Parales JC, Verdejo-García A, Billieux J (2012). Validation of a short Spanish version of the UPPS-P impulsive behavior scale. Trastor Adict.

[51] Zapolski TCB, Stairs AM, Settles RF, Combs JL, Smith GT (2010). The measurement of dispositions to rash action in children. Assessment.

[52] Coskunpinar A, Dir AL (2013). Cyders M a. Multidimensionality in impulsivity and alcohol use: a Meta-analysis using the UPPS model of impulsivity. Alcohol Clin Exp Res.

[53] Spillane NS, Smith GT, Kahler CW (2010). Impulsivity-like traits and smoking behavior in college students. Addict Behav.

[54] Cyders MA, Zapolski TCB, Combs JL, Settles RF, Fillmore MT, Smith GT (2010). Experimental effect of positive urgency on negative outcomes from risk taking and on increased alcohol consumption. Psychol Addict Behav.

[55] Michalczuk R, Bowden-Jones H, Verdejo-Garcia A, Clark L (2011). Impulsivity and cognitive distortions in pathological gamblers attending the UK National Problem Gambling Clinic: a preliminary report. Psychol Med.

[56] Simons JS, Maisto SA, Wray TB (2010). Sexual risk taking among young adult dual alcohol and marijuana users. Addict Behav.

[57] Zapolski TCB, Cyders MA, Smith GT (2009). Positive urgency predicts illegal drug use and risky sexual behavior. Psychol Addict Behav.

[58] Marmorstein NR (2013). Associations between dispositions to rash action and internalizing and externalizing symptoms in children. J Clin Child Adolesc Psychol.

[59] Dvorak RD, Pearson MR, Kuvaas NJ (2013). The five-factor model of impulsivity-like traits and emotional lability in aggressive behavior. Aggress Behav.

[60] Hecht LK, Latzman RD (2015). Revealing the nuanced associations between facets of trait impulsivity and reactive and proactive aggression. Pers Individ Dif..

[61] Taherifard M, Abolghasemi A, Hajloo N (2015). Positive and negative urgency and sleep quality among patients with borderline and antisocial personality disorders. Arch Psychiatry Psychother.

[62] Giovanelli A, Hoerger M, Johnson SL, Gruber J (2013). Impulsive responses to positive mood and reward are related to mania risk. Cogn Emot.

[63] Meyer TD, Newman AL, Jordan G (2015). Vulnerability for mania - is it linked to problems delaying gratification?. Psychiatry Res.

[64] Anestis MD, Tull MT, Lavender JM, Gratz KL (2014). The mediating role of non-suicidal self-injury in the relationship between impulsivity and suicidal behavior among inpatients receiving treatment for substance use disorders. Psychiatry Res.

[65] Peters JR, Upton BT, Baer RA (2013). Brief report: relationships between facets of impulsivity and borderline personality features. J Personal Disord.

[66] Delgado-Rico E, Río-Valle JS, González-Jiménez E, Campoy C, Verdejo-García A (2012). BMI predicts emotion-driven impulsivity and cognitive inflexibility in adolescents with excess weight. Obesity.

[67] Stojek MM, Fischer S, Murphy CM, MacKillop J (2014). The role of impulsivity traits and delayed reward discounting in dysregulated eating and drinking among heavy drinkers. Appetite.

[68] Cyders MA, Coskunpinar A, VanderVeen JD. In: Zeigler-Hill V, Marcus D, editors. Urgency - a common transdiagnostic endophenotype for maldaptive risk-taking: The Dark Side of Personality. Washington, DC: Americal Psycholgoical Association; 2016.

[69] Fischer S, Smith GT, Cyders MA (2008). Another look at impulsivity: a meta-analytic review comparing specific dispositions to rash action in their relationship to bulimic symptoms. Clin Psychol Rev.

[70] Fischer S, Smith GT (2008). Binge eating, problem drinking, and pathological gambling: linking behavior to shared traits and social learning. Pers Individ Dif..

[71] Dinc L, Cooper AJ (2015). Positive affective states and alcohol consumption: the moderating role of trait positive urgency. Addict Behav.

[72] Cyders MA, Smith GT (2010). Longitudinal validation of the urgency traits over the first year of college. J Pers Assess.

[73] Smith GT, Fischer S, Fister SM (2003). Incremental validity principles in test contruction. Psychol Assess.

[74] Telch CF, Agras WS (1996). Do emotional states influence binge eating in the obese?. Int J Eat Disord.

[75] Cyders MA, Dzemidzic M, Eiler WJ, Coskunpinar A, Karyadi K, Kareken DA (2014). Negative urgency and ventromedial prefrontal cortex responses to alcohol cues: FMRI evidence of emotion-based impulsivity. Alcohol Clin Exp Res.

[76] Karyadi KA, King KM (2011). Urgency and negative emotions: evidence for moderation on negative alcohol consequences. Pers Individ Dif..

[77] Pang RD, Farrahi L, Glazier S, Sussman S, Leventhal AM (2014). Depressive symptoms, negative urgency and substance use initiation in adolescents. Drug Alcohol Depend.

[78] Joormann J, Gotlib IH (2010). Emotion regulation in depression: relation to cognitive inhibition. Cogn Emot..

[79] Bylsma LM, Taylor-Clift A, Rottenberg J (2011). Emotional reactivity to daily events in major and minor depression. J Abnorm Psychol.

[80] McFarland BR, Klein DN (2009). Emotional reactivity in depression: diminished responsiveness to anticipated reward but not to anticipated punishment or to nonreward or avoidance. Depress Anxiety.

[81] Gruber J, Harvey AG, Johnson SL (2009). Reflective and ruminative processing of positive emotional memories in bipolar disorder and healthy controls. Behav Res Ther.

[82] Muhtadie L, Johnson SL, Carver CS, Gotlib IH, Ketter TA (2014). A profile approach to impulsivity in bipolar disorder: the key role of strong emotions. Acta Psychiatr Scand.

[83] Fergus TA, Bardeen JR (2014). Emotion regulation and obsessive–compulsive symptoms: a further examination of associations. J Obsessive Compuls Relat Disord.

[84] Axelrod SR, Perepletchikova F, Holtzman K, Sinha R (2011). Emotion regulation and substance use frequency in women with substance dependence and borderline personality disorder receiving dialectical behavior therapy. Am J Drug Alcohol Abuse.

[85] Villalta-Gil V, Meléndez-Pérez I, Russell T, Surguladze S, Radua J, Fusté M (2013). Functional similarity of facial emotion processing between people with a first episode of psychosis and healthy subjects. Schizophr Res.

[86] Habel U, Chechko N, Pauly K, Koch K, Backes V, Seiferth N (2010). Neural correlates of emotion recognition in schizophrenia. Schizophr Res.

[87] Yang C, Zhang T, Li Z, Heeramun-Aubeeluck A, Liu N, Huang N (2015). The relationship between facial emotion recognition and executive functions in first-episode patients with schizophrenia and their siblings. BMC Psychiatry.

[88] Barkl SJ, Lah S, Harris AWF, Williams LM (2014). Facial emotion identification in early-onset and first-episode psychosis: a systematic review with meta-analysis. Schizophr Res.

[89] Ammerman BA, Kleiman EM, Uyeji LL, Knorr AC, McCloskey MS (2015). Suicidal and violent behavior: the role of anger, emotion dysregulation, and impulsivity. Pers Individ Dif..

[90] Beck JS (2011). Cognitive behavior therapy: basics and beyond.

[91] Linehan MM (1993). Cognitive-behavioral treatment of borderline personality disorder.

[92] Kabat-Zinn J (2003). Mindfulness-based interventions in context: past, present, and future. Clin Psychol Sci Pract.

[93] Manasse SM, Espel HM, Schumacher LM, Kerrigan SG, Zhang F, Forman EM (2016). Does impulsivity predict outcome in treatment for binge eating disorder? A multimodal investigation. Appetite.

[94] Fox HC, Bergquist KL, Hong KI, Sinha R (2007). Stress-induced and alcohol cue-induced craving in recent abstinent alcohol-dependent individuals. Alcohol Clin Exp Res.

[95] Chester DS, Lynam DR, Milich R, Powell DK, Andersen AH, DeWall CN (2016). How do negative emotions impair self-control? A neural model of negative urgency. NeuroImage.

[96] Chester DS, Lynam DR, Milich R, DeWall CN (2016). Craving versus control: negative urgency and neural correlates of alcohol cue reactivity. Drug Alcohol Depend.

[97] VanderVeen JD, Hershberger AR, Cyders MA (2016). UPPS-P model impulsivity and marjiunana use behaviors in adolescents: a meta-analysis. Drug Alcohol Depend.

[98] Stautz K, Cooper A (2013). Impulsivity-related personality traits and adolescent alcohol use: a meta-analytic review. Clin Psychol Rev.

[99] Hulvershorn LA, Hummer TA, Fukunaga R, Leibenluft E, Finn P, Cyders MA (2015). Neural activation during risky decision-making in youth at high risk for substance use disorders. Psychiatry Res. Neuroimaging.

[100] Glenn CR, Klonsky ED (2010). A multimethod analysis of impulsivity in nonsuicidal self-injury. Pers Disord Theory Res Treat.

[101] Witte TK, Merrill KA, Stellrecht NE, Bernert RA, Hollar DL, Schatschneider C (2008). “Impulsive” youth suicide attempters are not necessarily all that impulsive. J Affect Disord.

[102] McKeown R, Garrison C, Cuffe S, Waller J, Jackson K, Addy C (1998). Incidence and predictors of suicidal behaviors in a longitudinal sample of young adolescents. J Am Acad Child Adolesc Psychiatry.

[103] Schönenberg M, Jusyte A (2014). Investigation of the hostile attribution bias toward ambiguous facial cues in antisocial violent offenders. Eur Arch Psychiatry Clin Neurosci.

[104] Fischer S, Smith GT (2004). Deliberation affects risk taking beyond sensation seeking. Pers Individ Dif.

[105] Koob GF, Volkow ND (2010). Neurocircuitry of addiction. Neuropsychopharmacology.

[106] Everitt BJ, Robbins TW (2005). Neural systems of reinforcement for drug addiction: from actions to habits to compulsion. Nat Neurosci.

[107] Kotov R, Krueger RF, Watson D, Achebach TM, Althoff RR, Bagby RM (2017). The hierarchical taxonomy of psychopathology (HiTOP): a dimensional alternative to traditional nosologies. J Abnorm Psychol.

[108] World Health Organization (1992). The ICD-10 classification of mental and behavioral disorders: clinical descriptions and diagnostic guidelines (Vol.1).

[109] Hershberger AR, Um M, Cyders MA (2017). The relationship between the UPPS-P impulsive personality traits and substance use psychotherapy outcomes: a meta -analysis. Drug Alcohol Depend.

[110] Zapolski TC, Settles RF, Cyders MA, Smith GT (2010). Borderline personality disorder, bulimia nervosa, antisocial personality disorder, ADHD, substance use: common threads, common treatment needs, and the nature of impulsivity. Indep Pract.

[111] Pearson CM, Wonderlich SA, Smith GT (2015). A risk and maintenance model for bulimia nervosa: from impulsive action to compulsive behavior. Psychol Rev.

[112] Savvidou LG, Fagundo AB, Fernández-Aranda F, Granero R, Claes L, Mallorquí-Baqué N (2017). Is gambling disorder associated with impulsivity traits measured by the UPPS-P and is this association moderated by sex and age?. Compr Psychiatry.

[113] Dir AL, Coskunpinar A, Cyders MA (2014). A meta-analytic review of the relationship between adolescent risky sexual behavior and impulsivity across gender, age, and race. Alcohol Clin Exp Res.

[114] Smith GT, Anderson KG, Monti PM, Colby SM, Tevyaw TAO (2001). Adolescent risk for alcohol problems as acquired preparedness: a model and suggestions for intervention. Adolescents, alcohol, and substance abuse: reaching teens through brief interventions.

[115] Kiluk BD, Nich C, Babuscio T, Carroll KM (2010). Quality versus quantity: acquisition of coping skills following computerized cognitive–behavioral therapy for substance use disorders. Addiction.

[116] Witkiewitz K, Donovan DM, Hartzler B (2012). Drink refusal training as part of a combined behavioral intervention: effectiveness and mechanisms of change. J Consult Cinical Psychol.

[117] Magill M, Kiluk BD, McCrady BS, Tonigan JS, Longabaugh R (2015). Active ingredients of treatment and client mechanisms of change in behavioral treatments for alcohol use disorders: progress 10 years later. Alcohol Clin Exp Res.

[118] Littlefield AK, Vergés A, Wood PK, Sher KJ (2012). Transactional models between personality and alcohol involvement: a further examination. J Abnorm Psychol.

[119] Littlefield AK, Sher KJ, Wood PK (2009). Is “maturing out” of problematic alcohol involvement related to personality change?. J Abnorm Psychol.

[120] Guller L, Zapolski TCB, Smith GT (2015). Personality measured in elementary school predicts middle school addictive behavior involvement. J Psychopathol Behav Assess.

[121] Conrod PJ, Castellanos-Ryan N, STrang J (2010). Brief, personality-targeted coping skills interventions and survival as a non–drug user over a 2-year period during adolescence. Arch Gen Psychiatry.

[122] Conrod PJ, Castellanos-Ryan N, Mackie C (2011). Long-term effects of a personality-targeted intervention to reduce alcohol use in adolescents. J Consult Cinical Psychol.

[123] Conrod PJ, O’Leary-Barrett M, Newton N, Topper L, Castellanos-Ryan N, Mackie C (2013). Effectiveness of a selective, personality-targeted prevention program for adolescent alcohol use and misuse. JAMA Psychiatry.

[124] Weiss NH, Tull MT, Davis LT, Searcy J, Williams I, Gratz KL (2015). A preliminary experimental investigation of emotion dysregulation and impulsivity in risky behaviours. Behav Chang.

[125] Zapolski TCB, Smith GT (2017). Pilot study: implementing a breif DBT skills program in schools to reduce health risk beahviors among early adolescents. J Sch Nurs.

[126] Amaro H, Magno-Gatmaytan C, Meléndez M, Cortés DE, Arevalo S, Margolin A (2010). Addiction treatment intervention: an uncontrolled prospective pilot study of spiritual self-schema therapy with Latina women. Subst Abus.

[127] Margolin A, Schuman-Olivier Z, Beitel M, Arnold RM, Fulwiler CE, Avants SK (2007). A preliminary study of spritual self-schema (3-S+) therapy for reducing impulsivity in HIV-positive drug users. J Clin Psychol.

[128] Littlefield AK, Stevens AK, Cunningham S, Jones RE, King KM, Schumacher JA (2015). Stability and change in multi-method measures of impulsivity across residential addictions treatment. Addict Behav.

[129] Reis AD, Castro LA, Faria R, Laranjeira R (2008). Craving decrease with topiramate in outpatient treatment for cocaine dependence: an open label trial. Rev Bras Psiquiatr.

[130] Blonigen DM, Timko C, Moos BS, Moos RH (2009). Treatment, alcoholics anonymous, and 16-year changes in impulsivity and legal problems among men and women with alcohol use disorders. J Stud Alcohol Drugs.

[131] Rinne T, van den Brink W, Wouters L, van Dyck R (2002). SSRI treatment of borderline personality disorder: a randomized, placebo-controlled clinical trial for female patients with borderline personality disorder. Am J Psychiatry.

[132] Kendall PC, Finch AJ (1978). A cognitive-behavioral treatment for impulsivity: a group comparison study. J Consult Clin Psychol.

[133] Kendall PC, Wilcox LE (1980). Cognitive-behavioral treatment for impulsivity: concrete versus conceptual training in non-self-controlled problem children. J Consult Clin Psychol.

[134] Aklin WM, Tull MT, Kahler CW, Lejuez CW (2009). Risk-taking propensity changes throughout the course of residential subsatnce abuse treatment. Pers Individ Dif..

[135] Gonçalves PD, Ometto M, Bechara A, Malbergier A, Amaral R, Nicastri S (2014). Motivational interviewing combined with chess accelerates improvement in executive functions in cocaine dependent patients: a one-month prospective study. Drug Alcohol Depend.

[136] Conrod PJ, O’Leary-Barrett M, Newton N, Topper L, Castellanos-Ryan N, Mackie C (2013). Effectiveness of a selective, personality-targeted prevention program for adolescent alcohol use and misuse: a cluster randomized controlled trial. JAMA Psychiatry.

[137] Zapolski TCB (2010). An intervention to improve goal-directed behavior and reduce rissy behaviors in early adolescents.

[138] Heinz AJ, Bui L, Thomas KM, Blonigen DM (2015). Distinct facets of impulsivity exhibit differential associations with substance use disorder treatment processes: a cross-sectional and prospective investigation among military veterans. J Subst Abus Treat.

[139] Santos-Ruiz A, Robles-Ortega H, Pérez-García M, Peralta-Ramírez MI (2017). Effects of the cognitive-behavioral therapy for stress management on executive function components. Spaninish J Psychol.

